# A269 THE MODULATION OF INTESTINAL INFLAMMATION BY PARABACTEROIDES GOLDSTEINII IN MURINE MODELS OF COLITIS

**DOI:** 10.1093/jcag/gwac036.269

**Published:** 2023-03-07

**Authors:** C Gerkins, M Oliero, R Hajjar, H Vennin Rendos, G Fragoso, A Calvé, K Diop, B Routy, M Santos

**Affiliations:** 1 Nutrition and Microbiome Laboratory, Institut du cancer de Montréal, Centre de recherche du Centre hospitalier de l’Université de Montréal (CRCHUM); 2 Digestive Surgery Service, CHUM; Faculty of Medicine, Université de Montréal; 3 Laboratory of Immunotherapy/Oncomicrobiome, CRCHUM; 4 Department of Medicine, Faculty of Medicine, Université de Montréal, Montréal, Canada

## Abstract

**Background:**

Inflammatory bowel diseases (IBDs), comprised mainly of Crohn’s disease and ulcerative colitis, are characterized by chronic and relapsing intestinal inflammation. Though the etiology of IBDs is not entirely understood, it is thought that an imbalance of the gut microbiota is one contributing factor, making it a potential target for the treatment of IBDs.

**Purpose:**

This study aims to examine how the potential probiotic *Parabacteroides goldsteinii* modulates intestinal inflammation in the context of experimental colitis.

**Method:**

Wild type C57BL/6 mice were divided into three groups. One group was given phosphate-buffered saline (PBS) via oral gavage without any further treatment. The second group was treated with 1% dextran sodium sulfate (DSS) for twelve days induce colitis and given PBS every three days. Finally, the third group was also treated with 1% DSS for twelve days and given *P. goldsteinii* via oral gavage every three days. Weight loss, stool consistency, and intestinal bleeding were monitored and were used to calculate the Disease Activity Index (DAI) daily. Postmortem, colon length was measured, and inflammation score was evaluated on hematoxylin and eosin-stained samples of the colon and spleen. Finally, interleukin (IL)-6 levels were measured in the colon tissue and the serum by ELISA.

**Result(s):**

Colonization was confirmed at day three after gavage by real-time PCR. Preliminary results show that DSS-treated mice lost significantly more weight by day twelve than mice colonized with *P. goldsteinii*. Additionally, on days eight and twelve, mice colonized with *P. goldsteinii* had significantly lower DAI scores than mice that received only PBS. Finally, mice colonized with *P. goldsteinii* had significantly longer colons than those that received only PBS.

**Conclusion(s):**

The preliminary data indicates that mice colonized with *P. goldsteinii* had less severe symptoms of colitis than mice only given PBS. This suggests that *P. goldsteinii* may attenuate inflammation in mice treated with DSS.

**Please acknowledge all funding agencies by checking the applicable boxes below:**

CIHR, Other

**Please indicate your source of funding;:**

ICM

**Disclosure of Interest:**

None Declared

